# Task Graph Generation for Heterogeneous UAV Swarms in Partially Observable Adversarial Environments

**DOI:** 10.3390/e28060708

**Published:** 2026-06-18

**Authors:** Wenxin Li, Yongxin Feng

**Affiliations:** School of Information Science and Engineering, Shenyang Ligong University, Shenyang 110158, China; liwenxin@sylu.edu.cn

**Keywords:** UAV swarm, task generation, task graph, partial observability, resource constraints

## Abstract

In partially observable adversarial environments, heterogeneous unmanned aerial vehicle (UAV) swarms must generate tasks online from noisy observations while respecting platform capabilities, consumable resources, and structural dependencies among tasks. This paper proposes a task graph generation method that converts local observations, target beliefs, and UAV resource states into executable task graphs with explicit resource semantics and inter-task relations. The method first constructs a sufficiently expressive candidate task graph in the belief and resource spaces. An offline search teacher then evaluates future trajectory particles, resource feasibility, and structural interaction values to produce supervision for node selection, marginal task value, and relation prediction. A relation-biased graph attention network learns to generate task graphs online, and a task manager further performs task filtering, dependency repair, conflict completion, and resource checking. Simulation results under complex observation pressure and unseen adversarial strategies show that the proposed method consistently improves structural generation quality and execution feasibility. Compared with Graphormer, it improves the task-graph utility, task-edge F1-score, and executable-graph ratio by 5.83%, 5.41%, and 2.68%, respectively, while reducing the infeasible-task ratio by 35.14%. These results indicate that combining an offline search teacher with resource-constrained graph modeling provides an effective front-end task organization mechanism for heterogeneous UAV swarm planning.

## 1. Introduction

Unmanned aerial vehicle (UAV) swarms offer flexible deployment, wide-area coverage, platform redundancy, and rapid task response. Their use has expanded from single-platform autonomous flight to multi-UAV perception, dynamic resource scheduling, and autonomous decision making in adversarial environments. Recent studies have investigated multi-UAV systems for target search, disaster response, communication relay, boundary protection, and low-altitude security. As task scale and environmental uncertainty increase, swarm coordination depends not only on the motion-control accuracy of individual UAVs, but also on whether the system forms stable task organizations under local observations, limited communication, and heterogeneous resources [1,2,3].

From the viewpoint of uncertainty-aware decision making, the key difficulty is not only to allocate a known list of tasks, but also to decide which tasks should exist when the state of the adversarial environment is incompletely observed. Target-mode beliefs, observation confidence, communication quality, and resource margins form an evolving information state. Therefore, the proposed problem is closely related to entropy-based uncertainty representation and information-theoretic decision making, where a lower-ambiguity belief supports more decisive interception or blocking actions, whereas a high-entropy belief should trigger confirmation, tracking, or communication-support tasks before committing scarce strike resources [4,5].

In a typical adversarial scenario, a blue heterogeneous UAV swarm must respond to red-side behaviors such as direct penetration, flank maneuvering, electronic jamming, and decoy deception. Unlike routine inspection, fixed-route coverage, or target tracking with a given target set, these missions are closed-loop and strategic: red targets adapt their motion patterns in response to the blue deployment and interception posture, while blue sensors are affected by occlusion, noise, and electronic interference. The resulting situation is only partially observed and is represented by local measurements, historical tracks, and target beliefs. Directly feeding such a situation into a conventional task allocator often leads to missing task nodes, incorrect relation edges, and resource-infeasible assignments. In realistic adversarial operations, tasks must be generated online as the situation evolves, and the quality of the generated tasks directly affects downstream cooperative allocation and execution.

Classical task allocation and resource-constrained mission planning provide the first methodological basis for this study. The classical consensus-based bundle algorithm (CBBA) solves multi-agent task allocation through distributed auctions and is effective when task rewards and constraints are explicitly available [6]. More general taxonomies of multi-robot task allocation emphasize that allocation algorithms usually start from an already defined set of tasks, agent capabilities, and constraints [7,8]. Recent work has introduced graph neural networks (GNNs), hybrid optimization, and reinforcement learning into UAV, unmanned ground vehicle, and multi-robot coordination. Examples include adaptive deep GNNs for UAV–CAV (connected and autonomous vehicle) dynamic task allocation [9]; decentralized graph-attention-based allocation for multi-robot target localization [10]; and joint trajectory, spectrum, routing, offloading, and computation optimization for UAV edge systems and communication networks [2,11]. These studies motivate our modeling of task resource requirements, communication constraints, and feasibility checks, but they mainly address allocation and scheduling after candidate tasks are already specified.

Domain studies on UAV mission planning and coverage further show that platform heterogeneity, energy limits, communication reachability, and task timing strongly influence mission feasibility [3,12]. Resource-constrained scheduling theory also provides useful models for precedence and renewable/nonrenewable resource feasibility [13]. However, many scheduling and defense-allocation formulations assume that the task set or precedence graph is already known before optimization begins. Defense-oriented adversarial missions differ from pure coverage or search tasks because red-side motion deliberately creates false priorities, jams sensing links, and induces resource exhaustion. A problem-driven task-generation formulation is therefore needed: the planner must expose the intermediate task structure, make the uncertainty state visible to the decision process, and preserve resource and dependency information before downstream allocation is attempted.

Partially observable planning, online replanning, and multi-agent reinforcement learning (MARL) constitute a second line of related work. Partially Observable Monte Carlo Planning (POMCP) approximates policies in large partially observable Markov decision processes (POMDPs) using sampling-based online search [14]. Such online search reacts to updated beliefs, but repeated expansion of a large task-structure search space is too expensive for frequent UAV replanning and does not by itself expose an editable task graph. Recent studies on privileged learning and asymmetric training further show how simulation-time access to global states provides useful supervision when execution must rely on local observations [15]. In MARL, graph-based information aggregation has been used to improve scalability [16]; centralized-training information has been used to guide individual exploration under sparse rewards [17]; scalable safe policy optimization has incorporated local interactions and safety constraints [18]; and communication-aware MARL has highlighted the importance of communication targets, contents, costs, and constraints under partial observability [19]. Recent multimodal learning and prompt-based fusion studies, including FMSA for few-shot multimodal sentiment analysis with integrated prompt learning and vision–language models, also indicate that reliable autonomous decision making increasingly depends on robust fusion under uncertain, heterogeneous observations [20,21,22]. These works inform our treatment of belief states, uncertainty, offline teacher search, and communication resources. However, their outputs are usually actions, policies, or value functions rather than explicit task structures containing precedence, support, and conflict relations.

Task graph generation occupies an intermediate level between direct task assignment and end-to-end policy learning. Compared with direct assignment, it explicitly represents missing prerequisites, support requirements, mutually exclusive tasks, and infeasible resource combinations before UAV teams are allocated. Compared with an end-to-end policy, it yields an interpretable and editable structure that supports repair, checking, and transfer to different downstream allocators. This is particularly useful in dynamic UAV scenarios, where the system must revise not only who executes a task, but also whether reconnaissance, relay, blocking, or interception tasks should be generated at all.

Relational graph modeling and graph generation provide a natural language for task-structure representation. Graph attention and coordination-graph models capture interactions among agents, tasks, and environmental entities. Hyper Graphical Attention Policy (HGAP) uses graph attention to satisfy permutation invariance and equivariance in partially observable multi-agent decision making [23], while Group-Aware Coordination Graph (GACG) infers coordination graphs from both individual relations and group dependencies [24]. Formal methods have also been used in dynamic multi-robot task allocation, where satisfiability modulo theories (SMT) solvers enforce completeness and constraint consistency [25]. These methods motivate graph-based task representation, yet generic graph generation models usually lack UAV-specific task types, resource semantics, time constraints, and downstream executability. Formal allocation methods, meanwhile, remain computationally stressed under severe uncertainty and large candidate-task combinations.

Graph diffusion models are a promising family of generative models because discrete-state continuous-time diffusion and latent graph diffusion jointly generate node attributes, edge attributes, and graph structure [26,27]. Nevertheless, existing graph diffusion methods are not directly suitable for the present online task-generation problem without substantial modification. First, task graphs are subject to hard executability constraints: a generated interception node requires prior identification under low confidence, sufficient strike resources, and a nonconflicting support chain. Unconstrained denoising produces graphs that are locally plausible but violate resource, precedence, or conflict rules. Second, diffusion inference typically requires multiple denoising steps, which is expensive for online replanning when the UAV swarm updates the graph after each observation cycle. Third, relational consistency is typed and semantic: a support edge, a precedence edge, and a conflict edge have different feasibility implications and cannot be treated as exchangeable adjacency entries. A diffusion-based extension requires typed node-edge noise processes, resource-aware conditional embeddings, constraint-guided denoising, projection or repair layers after each denoising step, and few-step distillation for online inference. This extension is feasible when the diffusion process itself incorporates resource semantics, task dependencies, and executability checks, whereas this paper focuses on a supervised relation-biased graph generator with an explicit task-management layer.

This paper studies task graph generation for heterogeneous UAV swarms in partially observable adversarial environments. The basic idea is to construct candidate task nodes and candidate relations from current observations, historical beliefs, and platform resources; distill supervision from an offline search teacher using future trajectories, resource feasibility, and structural value; and train a relation-biased graph network to generate executable task graphs online. The main contributions are as follows:

(1) A resource-constrained task graph model is established for heterogeneous UAV swarms under partial observability. The model unifies current observations, target beliefs, and UAV resource states as task-generation inputs and represents tasks with task types, resource requirements, deadlines, reward-risk attributes, and structural edges.
(2) An offline search teacher is proposed for task-graph supervision. Using simulated true states, future trajectory particles, and resource-feasibility evaluation, the teacher produces node-selection labels, marginal-value labels, and task-relation labels.
(3) A relation-biased graph generation network and an online task manager are designed. The network jointly encodes spatial, semantic, temporal, and resource relations among candidate tasks, while the task manager performs filtering, dependency repair, conflict completion, and resource checking.
(4) A task-generation-oriented experimental evaluation is constructed. The evaluation covers task-graph utility, node F1-score, edge F1-score, executable-graph ratio, graph edit distance, online latency, generalization to unseen adversarial strategies, tail risk, and module ablation.

## 2. System Model and Problem Formulation

### 2.1. Scenario and Notation

Consider a two-dimensional continuous mission area

(1) Ω=[0,L]×[0,L],

where *L* is the side length. The blue heterogeneous UAV swarm defends a boundary located at $x=x_{\partial }$. Red targets enter from the far side of the boundary and approach it through direct penetration, flank maneuvering, jamming suppression, or decoy deception. We use the saturation operator

(2) $${\left[z\right]}_{a}^{b}=\min\left\{\max\left\{z,a\right\},b\right\}.$$

The blue UAV set is

(3) $$U=\{u_{1},u_{2},\ldots ,u_{N}\},$$

and the active red target set is

(4) $$E_{t}=\left\{e_{1},e_{2},\ldots ,e_{M_{t}}\right\}.$$

The state of UAV $u_{i}$ at time *t* is

(5) $$x_{i}\left(t\right)=\left(p_{i}\left(t\right),v_{i},E_{i}\left(t\right),\zeta_{i},\alpha_{i},\beta_{i}\left(t\right),\nu_{i}\left(t\right)\right),$$

where $p_{i}\left(t\right)$ is the position, $v_{i}$ is the maximum speed, $E_{i}\left(t\right)$ is the remaining energy, $\zeta_{i}$ is the platform role, $\alpha_{i}$ is the non-consumable capability vector, $\beta_{i}\left(t\right)$ is the consumable resource vector, and $\nu_{i}\left(t\right)\in \left\{0,1\right\}$ denotes availability. The capability and resource vectors are

(6) $$\alpha_{i}={\left[\alpha_{i,1},\alpha_{i,2},\alpha_{i,3},\alpha_{i,4}\right]}^{⊤},$$

and

(7) $$\beta_{i}\left(t\right)={\left[\beta_{i,1}\left(t\right),\beta_{i,2}\left(t\right),\beta_{i,3}\left(t\right),\beta_{i,4}\left(t\right)\right]}^{⊤}.$$

The meanings of the four capability and resource dimensions are summarized in Table 1 and Table 2, respectively.

The true state of red target $e_{j}$ is

(8) $$y_{j}\left(t\right)=\left(s_{j}\left(t\right),{\dot{s}}_{j}\left(t\right),m_{j},\nu_{j}^{e}\left(t\right)\right),$$

where $s_{j}\left(t\right)$ is the position, ${\dot{s}}_{j}\left(t\right)$ is the velocity, $m_{j}$ is the target mode, and $\nu_{j}^{e}\left(t\right)$ is the target status. The mode set is

(9) M={1,2,3,4},

corresponding to direct penetration, flank maneuvering, jamming suppression, and decoy deception. The online generator cannot access $y_{j}\left(t\right)$; it is used only for simulation evaluation and offline teacher labeling.

**Figure 1:**
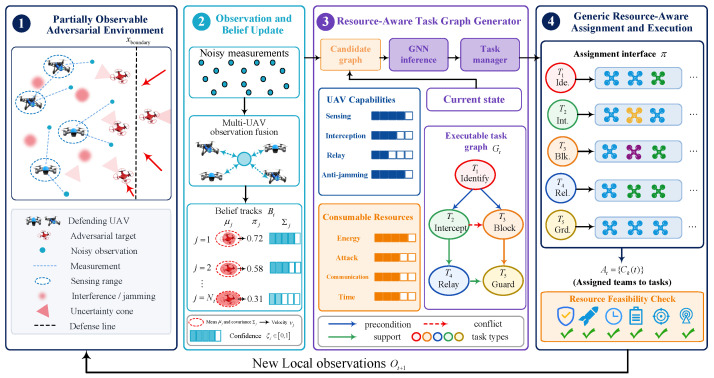
Observation, belief update, resource-constrained task graph generation, and cooperative execution in a partially observable adversarial scenario.

Figure 1 summarizes the closed-loop model. The UAV swarm starts from local observations, updates target beliefs, constructs candidate task graphs in the belief and resource spaces, outputs executable task graphs through task management, and passes them to a downstream cooperative allocation module.

### 2.2. Current Observation and Belief State

Because target maneuvers, occlusion, and jamming coexist, the blue side obtains only local noisy observations at time *t*. The observation of target $e_{j}$ by UAV $u_{i}$ is

(10) $$o_{ij}\left(t\right)=\left({\hat{s}}_{ij}\left(t\right),{\hat{m}}_{ij}\left(t\right),d_{ij}\left(t\right),\kappa_{i}\left(t\right)\right),$$

where ${\hat{s}}_{ij}\left(t\right)$ is a noisy position measurement, ${\hat{m}}_{ij}\left(t\right)$ is an imperfect mode cue, $d_{ij}\left(t\right)={∥p_{i}\left(t\right)-s_{j}\left(t\right)∥}_{2}$ is the relative distance, and $\kappa_{i}\left(t\right)$ is the local jamming intensity.

Let $E_{t}^{\kappa }$ denote the jamming target set. The jamming intensity at position p is

(11) $$\kappa \left(p,t\right)={\left[\sum_{e_{j}\in E_{t}^{\kappa }}\frac{R_{\kappa }-{∥p-s_{j}\left(t\right)∥}_{2}}{R_{\kappa }}\right]}_{0}^{1},$$

where $R_{\kappa }$ is the jamming radius. The effective detection range of UAV $u_{i}$ is

(12) $${\bar{R}}_{i}\left(t\right)=R_{i}\left[1-a_{\kappa }\kappa \left(p_{i}\left(t\right),t\right)\left(1-\alpha_{i,4}\right)\right],$$

where $R_{i}$ is the nominal detection range, $a_{\kappa }$ is the attenuation coefficient, and $\alpha_{i,4}$ is the anti-jamming capability. If $d_{ij}\left(t\right)\leq {\bar{R}}_{i}\left(t\right)$, the detection probability is

(13) $$P_{ij}\left(t\right)={\left[p_{0}-a_{p}\kappa_{i}\left(t\right)+b_{p}\alpha_{i,1}\right]}_{p_{\min}}^{p_{\max}}.$$

Once detected, the measured position satisfies

(14) $${\hat{s}}_{ij}\left(t\right)=s_{j}\left(t\right)+\varepsilon_{ij}\left(t\right),\;\varepsilon_{ij}\left(t\right)∼N\left(0,\sigma_{ij}^{2}\left(t\right)I\right),$$

with

(15) $$\sigma_{ij}\left(t\right)=\sigma_{0}+a_{\sigma }\kappa_{i}\left(t\right).$$

The raw observation set at time *t* is

(16) $$O_{t}=\left\{o_{ij}\left(t\right)|u_{i}\in U,e_{j}\in E_{t}\right\}.$$

To mitigate incomplete single-step observations, the system maintains target beliefs

(17) $$B_{t}=\left\{b_{1}\left(t\right),b_{2}\left(t\right),\ldots ,b_{M_{t}}\left(t\right)\right\}.$$

The belief of target $e_{j}$ is

(18) $$b_{j}\left(t\right)=\left(\mu_{j}\left(t\right),{\dot{\mu }}_{j}\left(t\right),\pi_{j}\left(t\right),\xi_{j}\left(t\right),\Sigma_{j}\left(t\right),t_{j}^{\ell }\right),$$

where $\mu_{j}\left(t\right)$ and ${\dot{\mu }}_{j}\left(t\right)$ are position and velocity estimates, $\pi_{j}\left(t\right)\in R^{4}$ is the mode-probability vector, $\xi_{j}\left(t\right)$ is the confidence, $\Sigma_{j}\left(t\right)$ is the uncertainty scale, and $t_{j}^{\ell }$ is the last observation time.

If $e_{j}$ is observed by UAV set $I_{j}\left(t\right)$, the fused position is

(19) $${\bar{o}}_{j}\left(t\right)=\frac{1}{|I_{j}\left(t\right)|}\sum_{i\in I_{j}\left(t\right)}{\hat{s}}_{ij}\left(t\right).$$

The velocity, confidence, and uncertainty are updated as

(20) $${\dot{\mu }}_{j}\left(t\right)=a_{v}\left[{\bar{o}}_{j}\left(t\right)-\mu_{j}\left(t-1\right)\right],$$

(21) $$\xi_{j}\left(t\right)=[\xi_{0}+a_{\xi }|I_{j}\left(t\right){\left|\right]}_{\xi_{\min}}^{\xi_{\max}},$$

and

(22) $$\Sigma_{j}\left(t\right)=\max\left\{\Sigma_{\min},\frac{\Sigma_{0}}{\sqrt{|I_{j}\left(t\right)|}}\right\}.$$

If the target is not observed, the belief is extrapolated over a finite horizon:

(23) $$\mu_{j}\left(t\right)=\mu_{j}\left(t_{j}^{\ell }\right)+{\dot{\mu }}_{j}\left(t_{j}^{\ell }\right)\min\left(t-t_{j}^{\ell },H_{b}\right),$$

(24) $$\xi_{j}\left(t\right)=\xi_{j}\left(t_{j}^{\ell }\right)a_{\ell }^{t-t_{j}^{\ell }},\;\Sigma_{j}\left(t\right)=\Sigma_{j}\left(t_{j}^{\ell }\right)+a_{\Sigma }\left(t-t_{j}^{\ell }\right).$$

The current observation state is therefore

(25) $$z_{t}=\left(O_{t},B_{t},{\left\{x_{i}\left(t\right)\right\}}_{i=1}^{N}\right).$$

### 2.3. Resource-Constrained Task Graph

At time *t*, the task structure is represented as a directed heterogeneous graph

(26) $$G_{t}=\left(V_{t},R_{t}\right),$$

where $V_{t}=\left\{\tau_{1},\tau_{2},\ldots ,\tau_{K_{t}}\right\}$ is the task-node set, and $R_{t}$ is the relation-edge set. Each task node is

(27) $$\tau_{k}=\left(y_{k},g_{k},w_{k},\rho_{k},\varrho_{k},T_{k},\psi_{k},\chi_{k},\iota_{k}\right),$$

where $y_{k}$ is the task type, $g_{k}$ is the target point, $w_{k}$ is the task reward, $\rho_{k}\in R^{4}$ is the non-consumable capability requirement, $\varrho_{k}\in R^{4}$ is the consumable resource requirement, $T_{k}$ is the deadline, $\psi_{k}$ is the risk, $\chi_{k}$ is the task confidence, and $\iota_{k}$ is the associated target index.

The task-type set is

(28) Y={1,2,3,4,5},

representing target identification, target interception, area blocking, communication support, and boundary guarding.

The resource semantics associated with these task types are summarized in Table 3.

A task-relation edge is

(29) $$r_{kl}=\left(\tau_{k},\tau_{l},\ell_{kl}\right),\;r_{kl}\in R_{t},$$

where $\ell_{kl}\in \left\{1,2,3\right\}$ is the relation type. Specifically, $\ell_{kl}=1$ denotes a precedence relation, $\ell_{kl}=2$ denotes a support relation, and $\ell_{kl}=3$ denotes a conflict relation.

**Figure 2:**
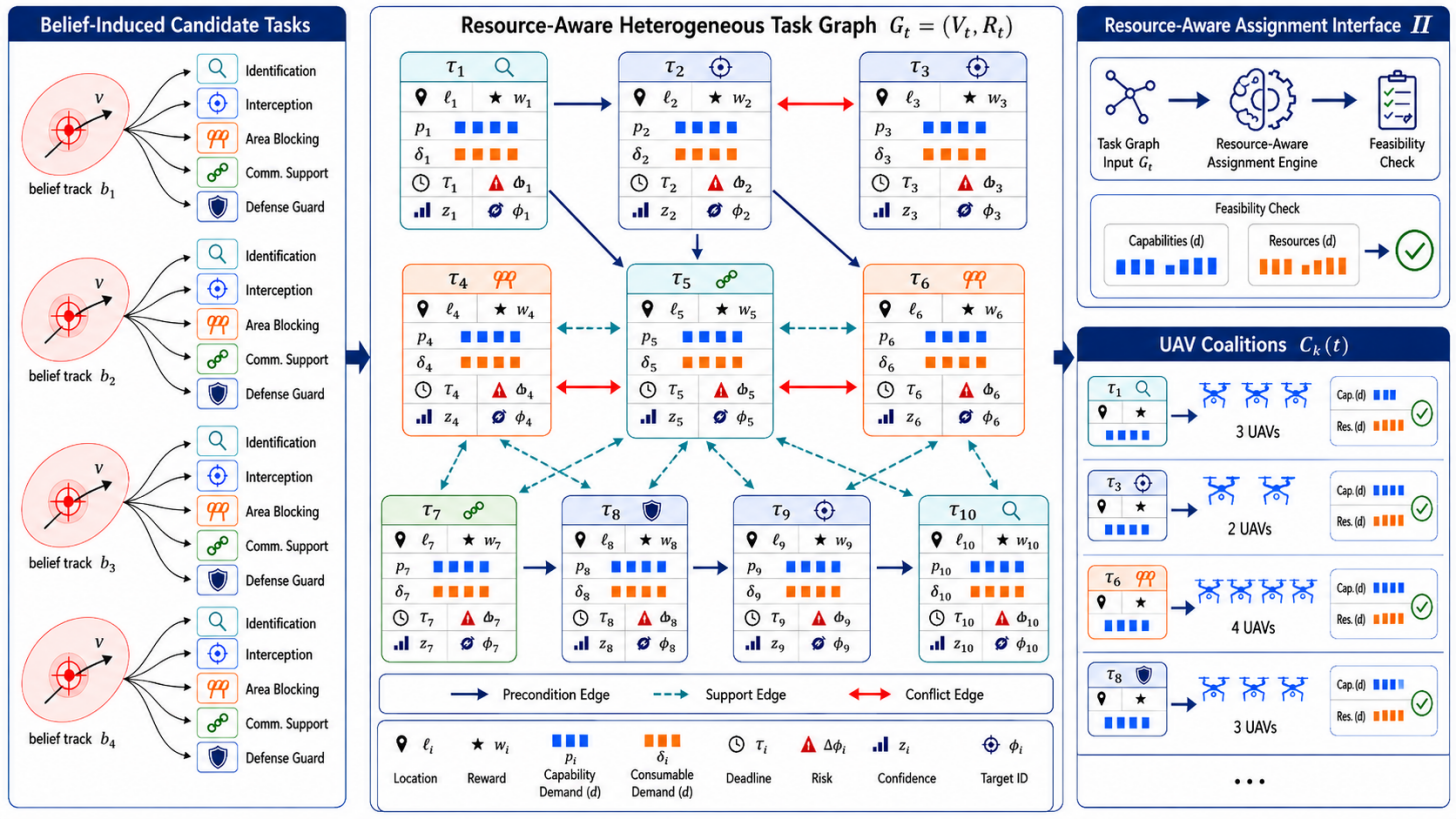
Node attributes, relation edges, and cooperative execution interface of the resource-constrained task graph.

Figure 2 illustrates the task-node attributes, typed relations, and cooperative execution interface of the resource-constrained task graph.

### 2.4. Execution Interface and Problem Statement

The downstream cooperative decision process is abstracted as a generic resource-aware allocation interface

(30) $$\Pi :\left(G_{t},{\left\{x_{i}\left(t\right)\right\}}_{i=1}^{N}\right)\mapsto A_{t},$$

where $A_{t}={\left\{C_{k}\left(t\right)\right\}}_{k=1}^{K_{t}}$ and $C_{k}\left(t\right)\subseteq U$ is the UAV team assigned to task $\tau_{k}$. The interface supports auction, matching, tree search, graph optimization, and multi-agent policy implementations; this paper does not bind the generator to a specific allocator.

For task $\tau_{k}$, the non-consumable capability constraint is

(31) $$\sum_{u_{i}\in C_{k}\left(t\right)}\alpha_{i,q}\geq \rho_{k,q},\;q=1,2,3,4.$$

The consumable resource constraint is

(32) $$\sum_{\tau_{k}:u_{i}\in C_{k}\left(t\right)}\Delta_{ik,h}\left(t\right)\leq \beta_{i,h}\left(t\right),\;h=1,2,3,4,$$

where $\Delta_{ik,h}\left(t\right)$ is the amount of resource type *h* consumed by UAV $u_{i}$ when executing task $\tau_{k}$.

The consumable-resource constraint uses a linear additive accounting model. This approximation is adopted because it gives the task manager and downstream allocator fast feasibility checks during online graph generation. It is most appropriate when the execution costs of different tasks are weakly coupled over a short planning window. In real UAV operations, resource consumption becomes nonlinear when task interactions are strong. Cooperative sensing and relay tasks create synergy and reduce marginal cost; simultaneous communication and sensing cause bandwidth and payload contention; and the order in which reconnaissance, relay, and interception tasks are executed changes both energy consumption and success probability. The proposed graph represents these interaction effects at the structural level through precedence, support, and conflict relations, while the resource-cost function remains additive. A natural extension is to replace the additive term by pairwise interaction costs, submodular resource models, sequence-dependent costs, and learned resource predictors calibrated from real flight or hardware-in-the-loop data.

Given the task graph and allocation, the structured execution value is

(33) $$J_{t}\left(G_{t},A_{t}\right)=\sum_{\tau_{k}\in V_{t}}J_{1}\left(\tau_{k},C_{k}\right)+\sum_{r_{kl}\in R_{t}}J_{2}\left(r_{kl},C_{k},C_{l}\right)-J_{3}\left(A_{t}\right),$$

where $J_{1}$ is the task-node execution value, $J_{2}$ is the relation value, and $J_{3}$ is the resource and load cost. The goal is to learn an observation-conditioned task graph generation policy

(34) $$\pi_{\theta }:z_{t}\mapsto G_{t},$$

that maximizes the long-term expected return when coupled with the allocation interface:

(35) $$\max_{\theta }E\left[\sum_{t=0}^{T}\gamma^{t}R\left(y_{t},G_{t},A_{t}\right)\right].$$

The single-step reward is

(36) $$R_{t}=\omega_{1}n_{t}^{+}-\omega_{2}n_{t}^{-}-\omega_{3}n_{t}^{0}-\omega_{4}e_{t}-\omega_{5}d_{t}-\omega_{6}o_{t}+\omega_{7}s_{t},$$

where $n_{t}^{+}$ is the number of successful interceptions, $n_{t}^{-}$ is the number of boundary breakthroughs, $n_{t}^{0}$ is the number of remaining active targets, $e_{t}$ is energy consumption, $d_{t}$ is response latency, $o_{t}$ is task-load cost, and $s_{t}$ is the task-success statistic.

## 3. Proposed Method

The proposed method takes the current observation state as input, constructs a candidate task graph, uses an offline search teacher to produce structured supervision, and trains a relation-biased graph attention network (GAT) for online task graph generation. It is designed for dynamic adversarial scenarios in which rule-based triggers cannot reliably cover the required task set.

### 3.1. Overall Framework

**Figure 3:**
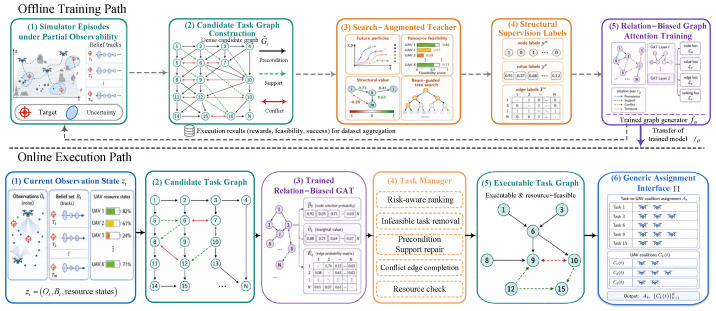
Overall framework of the proposed task graph generation method.

Figure 3 shows the overall framework. During offline training, simulated partially observable trajectories are used to construct dense candidate task graphs from target beliefs. The search teacher evaluates candidate subgraphs using future trajectory particles, resource feasibility, and task relations, and then produces node, value, and edge labels. The relation-biased graph attention network is trained on these labels. During online deployment, the trained model generates a task graph from the current observation state, and the task manager filters, repairs, checks, and sends the graph to the execution interface.

Let the current observation state at time *t* be $z_{t}$ and the candidate task graph be

(37) $${\tilde{G}}_{t}=\left({\tilde{V}}_{t},{\tilde{R}}_{t}\right).$$

The relation-biased graph attention network learns

(38) $$f_{\theta }:\left({\tilde{G}}_{t},z_{t}\right)\mapsto \left({\hat{p}}_{t},{\hat{v}}_{t},{\hat{E}}_{t}\right),$$

where ${\hat{p}}_{t}$ contains candidate-node selection probabilities, ${\hat{v}}_{t}$ contains marginal task values, and ${\hat{E}}_{t}$ is the relation probability matrix. The final task graph is produced by the task-management operator

(39) $$G_{t}=\Gamma \left({\tilde{G}}_{t},{\hat{p}}_{t},{\hat{v}}_{t},{\hat{E}}_{t}\right).$$

Algorithm 1 defines the input–output flow among four modules. The belief update module maps raw observations and UAV states into target beliefs $B_{t}$. The candidate graph constructor maps $B_{t}$ and resource states into ${\tilde{G}}_{t}$. The graph generator maps node and relation features into node-selection, value, and edge probabilities. The task manager then converts these probabilities into an executable graph by filtering, repairing, and completing relations, after which the generic execution policy returns assignments and resource updates for the next observation cycle.
**Algorithm 1** Observation-Conditioned Online Task Graph Generation1:**Input:** raw observations $O_{t}$; UAV states ${\left\{x_{i}\left(t\right)\right\}}_{i=1}^{N}$; trained generator $f_{\theta }$; task manager Γ2:**Output:** executable task graph $G_{t}$; assignment result $A_{t}$3:*Stage 1 – Belief update and candidate graph construction*4:Fuse raw observations $O_{t}$ and update belief tracks $B_{t}$5:Construct candidate task nodes ${\tilde{V}}_{t}$ from each belief track6:Construct initial relations ${\tilde{R}}_{t}$ using precedence, support, and conflict semantics7:Form ${\tilde{G}}_{t}=\left({\tilde{V}}_{t},{\tilde{R}}_{t}\right)$8:*Stage 2 – Graph inference*9:Build node feature matrix $X_{t}$ and pairwise relation tensor $R_{t}$10:$\left({\hat{p}}_{t},{\hat{v}}_{t},{\hat{E}}_{t}\right)\leftarrow f_{\theta }\left(X_{t},R_{t}\right)$11:*Stage 3 – Task graph management*12:Rank candidate tasks using ${\hat{p}}_{t}$, ${\hat{v}}_{t}$, and task risk13:Remove infeasible or redundant tasks according to resource constraints and per-target budgets14:Repair missing precedence and support tasks when high-confidence relations exist15:Add conflict edges for redundant attack tasks on the same target16:Obtain $G_{t}=\Gamma \left({\tilde{G}}_{t},{\hat{p}}_{t},{\hat{v}}_{t},{\hat{E}}_{t}\right)$17:*Stage 4 – Generic collaborative execution*18:Compute $A_{t}\leftarrow \Pi \left(G_{t},{\left\{x_{i}\left(t\right)\right\}}_{i=1}^{N}\right)$19:Execute assigned tasks and update UAV resources20:**return** $G_{t}$, $A_{t}$

### 3.2. Candidate Task Graph Construction and Task Management

Candidate construction aims to generate a sufficiently rich but size-controlled task set under the current belief state. For each target belief $b_{j}\left(t\right)$, the boundary urgency is

(40) $$u_{j}\left(t\right)=1-{\left[\frac{x_{\partial }-\mu_{j,x}\left(t\right)}{D_{\max}}\right]}_{0}^{1},$$

where $\mu_{j,x}\left(t\right)$ is the estimated target abscissa, and $D_{\max}$ is a normalization distance. The operator ${\left[z\right]}_{0}^{1}$ is the saturation operator defined in Section 2.1. Here, $x_{\partial }$ denotes the defended boundary, and $D_{\max}$ converts the distance-to-boundary term into a dimensionless urgency score. Therefore, $u_{j}\left(t\right)$ approaches one when the estimated target position $\mu_{j,x}\left(t\right)$ is close to the defended boundary and approaches zero when the target remains far from it. The attack tendency is

(41) $$a_{j}\left(t\right)=\pi_{j,1}\left(t\right)+\lambda_{a}\pi_{j,2}\left(t\right),$$

The two mode probabilities are weighted differently because direct penetration and flank maneuvering imply different urgency. The component $\pi_{j,1}\left(t\right)$ corresponds to a direct approach toward the defended boundary and is therefore assigned full weight, whereas $\pi_{j,2}\left(t\right)$ corresponds to a flank maneuver whose threat depends on lateral position and remaining time. The coefficient $\lambda_{a}$ downweights or upweights this second mode according to validation performance. and the overall risk is

(42) $$\psi_{j}\left(t\right)={\left[b_{1}u_{j}\left(t\right)+b_{2}a_{j}\left(t\right)+b_{3}\xi_{j}\left(t\right)+b_{4}\frac{\Sigma_{j}\left(t\right)}{\Sigma_{\max}}\right]}_{0}^{1}.$$

The coefficients $b_{1},b_{2},b_{3},b_{4}$ are nonnegative risk-aggregation weights fixed before testing. In the implementation, they are selected on validation episodes so that boundary urgency, attack tendency, belief confidence, and uncertainty scale contribute comparable normalized magnitudes to $\psi_{j}\left(t\right)$. The risk score is used for candidate ranking and pruning rather than as an isolated decision rule, which reduces sensitivity to any single coefficient; nevertheless, the uncertainty and resource ablations in Section 4.12 examine whether the generator remains effective when these risk-related inputs or constraints are removed.

For each target, the generator constructs five candidate tasks:

(43) $${\tilde{V}}_{j}\left(t\right)=\left\{\tau_{j,1},\tau_{j,2},\tau_{j,3},\tau_{j,4},\tau_{j,5}\right\},$$

corresponding to target identification, target interception, area blocking, communication support, and boundary guarding. The five candidates are not all forced into the final graph. They form a fixed semantic menu for each active target: identification reduces belief ambiguity, interception acts on high-threat targets, blocking creates a defensive spatial barrier, communication support mitigates jamming or link loss, and boundary guarding protects the defended line when target intent is uncertain. The subsequent pruning and task-management stages remove candidates whose risk, confidence, reward, or resource feasibility is insufficient. Their target points are determined by target beliefs and task semantics:

(44) $$g_{j,1}\left(t\right)=\mu_{j}\left(t\right),$$

(45) $$g_{j,2}\left(t\right)=\left({\left[\mu_{j,x}\left(t\right)+l_{j}\left(t\right)\right]}_{0}^{x_{\partial }-\epsilon },\mu_{j,y}\left(t\right)\right),$$

(46) $$g_{j,3}\left(t\right)=\left({\left[\frac{\mu_{j,x}\left(t\right)+x_{\partial }}{2}\right]}_{x_{\min}}^{x_{\partial }-\epsilon },\mu_{j,y}\left(t\right)\right),$$

(47) $$g_{j,4}\left(t\right)=\left({\left[\mu_{j,x}\left(t\right)+l_{4}\right]}_{x_{4}^{-}}^{x_{4}^{+}},{\left[\mu_{j,y}\left(t\right)\right]}_{y^{-}}^{y^{+}}\right),$$

(48) $$g_{j,5}\left(t\right)=\left(x_{\partial }-l_{5},{\left[\mu_{j,y}\left(t\right)\right]}_{y^{-}}^{y^{+}}\right).$$

The boundary clipping in the interception and blocking points prevents generated target points from crossing the defended boundary. The small margin ϵ>0 keeps the generated point strictly inside the feasible defense side, which avoids numerical ambiguity when a target is estimated exactly on the boundary. The bounds $x_{\min}$, $x_{4}^{-}$, $x_{4}^{+}$, $y^{-}$, and $y^{+}$ define the feasible spatial ranges for blocking and communication-support tasks.

Candidate rewards, deadlines, and resource requirements are

(49) $$w_{k}=w_{0}\left(y_{k}\right)+c_{1}\left(y_{k}\right)\psi_{k}+c_{2}\left(y_{k}\right)\chi_{k}+c_{3}{\left(y_{k}\right)}^{⊤}\pi_{\iota_{k}}\left(t\right),$$

(50) $$T_{k}=t+\left\lceil T_{\max}\left(y_{k}\right)-u_{\iota_{k}}\left(t\right)\left[T_{\max}\left(y_{k}\right)-T_{\min}\left(y_{k}\right)\right]\right\rceil ,$$

and

(51) $$\rho_{k}=\rho^{0}\left(y_{k}\right)+\psi_{k}\rho^{\psi }\left(y_{k}\right),\;\varrho_{k}=\varrho^{0}\left(y_{k}\right)+u_{\iota_{k}}\left(t\right)\varrho^{u}\left(y_{k}\right).$$

Relation edges are initialized from task semantics. A precedence edge is added when one task supplies a necessary precondition for another task; for example, target identification should precede interception when the belief confidence is below the attack threshold. A support edge is added when one task improves the feasibility or reliability of another task; for example, a communication-support task supports interception or blocking under strong jamming or weak connectivity. A conflict edge is added when two tasks compete for the same target, time window, spatial sector, or scarce strike resource. The candidate graph is then pruned by risk, reward, confidence, and resource feasibility to retain at most ${\tilde{K}}_{\max}$ task nodes. Redundant tasks are removed when they cover the same target with lower value and no additional support role, whereas infeasible tasks are removed when no available UAV coalition satisfies their capability and consumable-resource requirements.

The online task manager converts model outputs into an executable task graph. For task $\tau_{k}$, the selection score is

(52) $$s_{k}=a_{1}{\hat{p}}_{k}+a_{2}{\hat{v}}_{k}+a_{3}\psi_{k}+a_{4}\chi_{k}-a_{5}\Delta_{k},$$

where $\Delta_{k}$ is the infeasibility penalty relative to current resources. The manager selects tasks according to $s_{k}$ while enforcing the number constraint, per-target task budgets, precedence repair, support repair, and conflict-edge completion.

The repair mechanism is rule-guided but score-aware. If an interception task for target $e_{j}$ is retained while a required identification task is absent and the belief confidence $\xi_{j}\left(t\right)$ is below the confirmation threshold, the manager first attempts to insert the missing identification task. If the resource budget cannot support both tasks, the manager either removes the interception task or downgrades it to blocking or tracking according to the selection scores. Similarly, when strong jamming makes an attack chain depend on communication support, a missing support task is inserted if it has a feasible UAV coalition; otherwise, the unsupported high-risk task is penalized or removed. This repair step prevents locally high-scoring nodes from forming logically incomplete task chains.

### 3.3. Offline Search Teacher and Graph Generation Network

**Figure 4:**
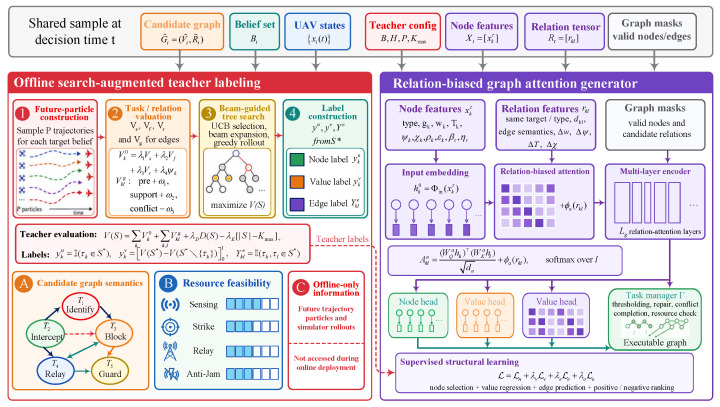
Offline search teacher and relation-biased graph attention generation network.

Figure 4 illustrates the search teacher and the graph generation network. The teacher samples future trajectory particles in belief space; evaluates future coverage, resource feasibility, and structural interaction values; and searches for high-value task subgraphs. The graph generator learns node, value, and edge labels from the teacher.

The offline teacher and the online generator use different information sets, as summarized in Table 4. The trained generator receives only the observation-conditioned belief state, candidate task graph, and current UAV resource states. Privileged simulator variables are used only for offline labeling, validation, and evaluation and are not provided to $f_{\theta }$ during online deployment.

The teacher first samples *P* future trajectories of horizon *H* for each active target:

(53) $$P_{j}={\left\{s_{j,p}^{0:H}\right\}}_{p=1}^{P}.$$

For candidate task $\tau_{k}$, the future-coverage value is

(54) $$V_{k}^{f}=\frac{1}{P}\sum_{p=1}^{P}\varphi \left(g_{k},s_{\iota_{k},p}^{0:H},y_{k}\right).$$

The resource-feasibility value is

(55) $$V_{k}^{r}=\max_{C\subseteq U,|C|\leq C_{\max}}\left[\frac{1}{4}\sum_{q=1}^{4}\min\left(1,\frac{\sum_{u_{i}\in C}\alpha_{i,q}}{\rho_{k,q}+\epsilon }\right)-\lambda_{d}\bar{d}\left(C,g_{k}\right)-\lambda_{b}\bar{b}\left(C,\tau_{k}\right)\right],$$

where $\bar{d}\left(C,g_{k}\right)$ is the average team arrival cost, and $\bar{b}\left(C,\tau_{k}\right)$ is the consumable resource cost. The task base value is

(56) $$V_{k}^{0}=\lambda_{1}V_{k}^{s}+\lambda_{2}V_{k}^{f}+\lambda_{3}V_{k}^{r}+\lambda_{4}\psi_{k},$$

where $V_{k}^{s}$ is the task semantic value.

For a task pair $\left(\tau_{k},\tau_{l}\right)$, the structural interaction value is

(57) $$V_{kl}^{g}=\left\{\begin{array}{cc} \omega_{1}, & \ell_{kl}=1,\\ \omega_{2}, & \ell_{kl}=2,\\ -\omega_{3}, & \ell_{kl}=3,\\ 0, & \mathrm{otherwise}. \end{array}\right.$$

Given a task subset S, the teacher evaluation function is

(58) $$V\left(S\right)=\sum_{\tau_{k}\in S}V_{k}^{0}+\sum_{\tau_{k},\tau_{l}\in S,k\neq l}V_{kl}^{g}+\lambda_{\Delta }D\left(S\right)-\lambda_{K}\left[|S\right|-K_{\max}{]}_{0}^{+\infty }.$$

Here, D(S) encourages coverage of diverse targets and task types. The teacher searches for a high-value subset $S^{★}$ through beam-limited Monte Carlo tree search and generates supervision accordingly.

Algorithm 2 summarizes the offline search-teacher labeling procedure.
**Algorithm 2** Offline Search-Teacher Labeling1:**Input:** candidate graph ${\tilde{G}}_{t}$; belief set $B_{t}$; UAV resource states; search budget *B*2:**Output:** node labels $y^{n}$; value labels $y^{v}$; edge labels $Y^{e}$3:Construct future trajectory particles $P_{j}$ for each active target belief4:**for** each candidate task $\tau_{k}\in {\tilde{V}}_{t}$ **do**5:   Compute semantic, future-coverage, and resource-feasibility values6:   Construct task base value $V_{k}^{0}$7:**end for**8:**for** each candidate relation $\left(\tau_{k},\tau_{l},\ell_{kl}\right)\in {\tilde{R}}_{t}$ **do**9:   Compute structural interaction value $V_{kl}^{g}$10:**end for**11:Search for the best subset $S^{★}$ using beam-guided tree search12:**for** each candidate task $\tau_{k}\in {\tilde{V}}_{t}$ **do**13:   Set $y_{k}^{n}\leftarrow I\left(\tau_{k}\in S^{★}\right)$14:   Set $y_{k}^{v}\leftarrow {\left[V\left(S^{★}\right)-V\left(S^{★}\setminus \left\{\tau_{k}\right\}\right)\right]}_{0}^{1}$15:**end for**16:**for** each candidate relation $\left(\tau_{k},\tau_{l},\ell_{kl}\right)\in {\tilde{R}}_{t}$ **do**17:   Set $Y_{kl}^{e}\leftarrow I\left(\tau_{k},\tau_{l}\in S^{★}\right)$18:**end for**19:**return** $y^{n}$, $y^{v}$, $Y^{e}$

For each candidate task $\tau_{k}$, the node feature is

(59) $$x_{k}^{\tau }=\left[e\left(y_{k}\right),g_{k},w_{k},T_{k},\psi_{k},\chi_{k},\rho_{k},\varrho_{k},b_{\iota_{k}}\left(t\right),\eta_{t}\right],$$

where $e(y_{k})$ is the task-type embedding, and $\eta_{t}$ is the team resource context. For task pair $\left(\tau_{k},\tau_{l}\right)$, the relation feature is

(60) $$\begin{array}{cc} r_{kl}= & [I_{k=l},I_{\iota_{k}=\iota_{l}},I_{y_{k}=y_{l}},d_{kl},\exp\left(-\lambda_{d}d_{kl}\right),I_{\ell_{kl}=1},\\ \; & I_{\ell_{kl}=2},I_{\ell_{kl}=3},w_{k}-w_{l},\psi_{k}-\psi_{l},T_{k}-T_{l},\chi_{k}-\chi_{l}]. \end{array}$$

The model embeds node features into the hidden space:

(61) $$h_{k}^{0}=\Phi_{\mathrm{in}}\left(x_{k}^{\tau }\right).$$

For attention head *a* at layer *l*, the relation-biased attention is

(62) $$A_{kl}^{a}=\frac{{\left(W_{Q}^{a}h_{k}\right)}^{⊤}\left(W_{K}^{a}h_{l}\right)}{\sqrt{d_{a}}}+\phi_{a}\left(r_{kl}\right),$$

(63) $${\bar{A}}_{kl}^{a}=\frac{\exp(A_{kl}^{a})}{\sum_{l^{\prime }}\exp\left(A_{kl^{\prime }}^{a}\right)}.$$

After multiple encoding layers, the outputs are

(64) $${\hat{p}}_{k}=\sigma \left(\Phi_{n}\left(h_{k}\right)\right),\;{\hat{v}}_{k}=\sigma \left(\Phi_{v}\left(h_{k}\right)\right),$$

(65) $${\hat{E}}_{kl}=\sigma \left(\Phi_{e}\left(\left[h_{k},h_{l},r_{kl}\right]\right)\right).$$

The training loss is

(66) $$L=L_{n}+\lambda_{v}L_{v}+\lambda_{e}L_{e}+\lambda_{o}L_{o},$$

where the first three terms are node-selection, value-regression, and edge-prediction losses. The final term is a ranking loss:

(67) $$L_{o}=\frac{1}{|P^{+}\left|\right|P^{-}|}\sum_{i\in P^{+}}\sum_{j\in P^{-}}\log\left[1+\exp(\Delta -z_{i}+z_{j})\right],$$

where $P^{+}$ is the teacher-selected task set, $P^{-}$ is the unselected set, and $z_{i}$ is the node-selection logit.

Algorithm 3 summarizes the training procedure of the relation-biased task graph generator.
**Algorithm 3** Training of the Relation-Biased Task Graph Generator1:**Input:** simulator episodes; candidate task factory; search teacher; graph generator $f_{\theta }$; iterations *I*; batch size *M*2:**Output:** trained generator parameters θ3:**for** each simulator episode **do**4:   **for** each decision step *t* **do**5:     Build current observation state $z_{t}$ and candidate graph ${\tilde{G}}_{t}$6:     Query the search teacher to obtain $\left(y^{n},y^{v},Y^{e}\right)$7:     Store the labeled graph sample in dataset D8:   **end for**9:**end for**10:**for** i=1 *I* **do**11:   Sample a mini-batch of *M* graph samples from D12:   Build padded node features, relation tensors, and graph masks13:   Predict node probabilities, values, and edge probabilities with $f_{\theta }$14:   Compute node, value, edge, and ranking losses15:   Update θ by gradient descent16:**end for**17:Tune task-selection thresholds, maximum task count, and per-target task budget on validation episodes18:**return** θ

### 3.4. Complexity and Method Characteristics

Let $\tilde{K}$ be the number of candidate tasks, *d* the hidden dimension, and $L_{g}$ the number of graph attention layers. The main cost of the relation-biased graph attention network comes from pairwise task encoding, and a forward pass has complexity

(68) $$O(L_{g}{\tilde{K}}^{2}d).$$

Candidate-graph pruning bounds online inference cost before the network forward pass. The search teacher is used only for offline labeling; its cost is controlled by limiting the search budget, caching future trajectory particles, and applying beam pruning.

## 4. Experiments and Analysis

### 4.1. Experimental Setup

The experiments are conducted in a two-dimensional adversarial defense area of 1000m×1000m. The blue side consists of a heterogeneous UAV swarm, while red targets approach the boundary using direct penetration, flank maneuvering, electronic jamming, decoy deception, and mixed strategies. UAVs form current belief states from local sensing, communication links, and historical observations; the task generator then outputs task nodes, relation edges, resource requirements, and executability labels. In each episode, the number of targets, observation-missing rate, jamming intensity, decoy ratio, remaining resources, and communication reachability are randomly sampled to avoid overfitting to fixed scripted scenarios.

Training uses multiple random seeds. Each seed corresponds to independent initial situations, target-behavior perturbations, and communication-noise sequences. During testing, all methods are evaluated under the same fixed seed set. Distributional experiments further sample different combinations of target scale and observation pressure and report mean, standard deviation, 5% quantile, median, and 95% quantile. The experiments are performed on Windows 11. The neural networks are implemented in PyTorch 2.11.0, with training and inference conducted on an NVIDIA RTX 4090 graphics processing unit (GPU).

For paired comparisons, all methods are evaluated on the same scenario seeds and the same generated target-condition sequences. Unless otherwise specified, distributional figures aggregate 1500 final-test task-graph samples. Paired win rates are computed episode by episode or sample by sample under matched conditions. For scalar paired metrics, the Wilcoxon signed-rank test is used because the latency and infeasibility distributions are not assumed to be Gaussian; Holm–Bonferroni correction is applied across pairwise method comparisons. Effect sizes are reported as standardized mean differences, and 95% confidence intervals are estimated by bootstrap resampling over paired samples. In the text, an improvement is treated as practically meaningful only when it is consistent across the primary metrics and does not create an unacceptable latency or infeasibility increase.

The experimental results are reported through integrated multi-panel figures that group related metrics by scenario dimension, uncertainty condition, and statistical quantity. Per-seed training traces and intermediate sensitivity plots are excluded from the main text because the primary comparisons are represented by distributional summaries, response surfaces, and paired statistical evidence.

The main simulation parameters and their ranges are summarized in Table 5.

The experiments are organized along four scenario dimensions: swarm size, target scale, uncertainty level, and computational-time focus. The final-test comparison aggregates standard adversarial episodes with 8∼32 UAVs and 6∼48 targets; the response-surface analysis isolates increasing target scale and observation pressure; the strong-jamming and decoy-deception analyses stress support-edge recovery, belief entropy, false-edge filtering, and tail risk; and the OOD experiment evaluates unseen flank, jamming, decoy, and mixed strategies. The implementation settings for these experiments are summarized in Table 6, while the offline wall-clock costs are reported subsequently.

Table 6 reports the implementation-critical settings for the simulator, teacher-labeling process, generator training, evaluation protocol, task manager, and baseline tuning.

**Figure 5:**
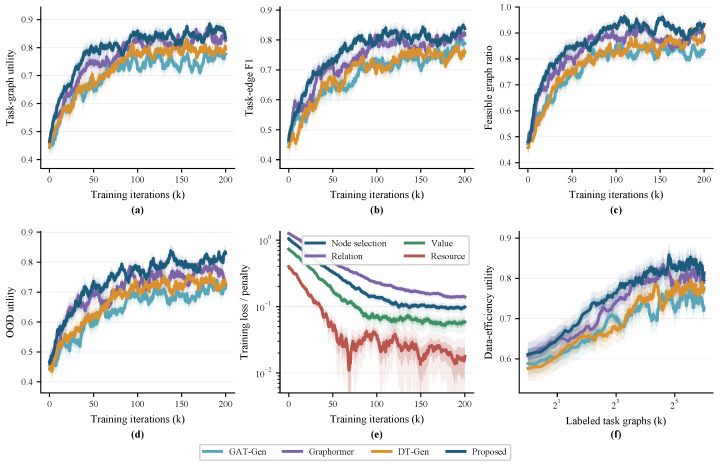
Training dynamics against strong baselines: (**a**) task-graph utility; (**b**) task-edge F1; (**c**) executable-graph ratio; (**d**) out-of-distribution (OOD) utility under unseen adversarial strategies; (**e**) node-selection, relation-prediction, value-estimation, and resource-penalty losses; (**f**) labeled task graphs and data-efficiency utility.

The wall-clock cost of data collection, teacher labeling, and neural-network training is reported by pipeline stage in Table 7. The table separates workload, wall-clock time, and computing device for the offline preparation pipeline. Online inference latency is reported in Table 8; the subsequent Figures are introduced and discussed in numerical order, beginning with Figure 5.

Six classes of metrics are used. Task-graph utility measures the overall value under threat coverage, reward–risk balance, deadline constraints, and resource cost. Task-node F1-score and task-edge F1-score evaluate whether task nodes and relation edges are correctly generated. The executable-graph ratio measures whether a generated graph passes resource, temporal, and logical consistency checks, while the infeasible-task ratio measures the fraction of task nodes that cannot be satisfied by any feasible UAV team. Graph edit distance measures the node and edge changes required to transform a generated graph into the teacher graph. Online generation latency measures the time from receiving the current observation to outputting the task graph.

### 4.2. Baselines and Adaptation

Five strong baselines are selected, with recent applications in task planning, task allocation, or task generation used as references for adaptation. CBBA follows the Clustering-CBBA idea for heterogeneous multi-UAV task allocation [28]; candidate tasks under the current observation are treated as bundle elements, and bids are constructed from reward, distance, and residual resources. Because this family is mainly designed for task allocation, relation edges are completed according to execution order, resource competition, and temporal conflicts. GAT-based generation (GAT-Gen) follows graph-attention-based unsupervised multi-robot task allocation [29]; it takes candidate task nodes and UAV resource embeddings as inputs and outputs task structures through node and edge heads. Graphormer follows the use of GNNs and Transformers in UAV–CAV mission planning [30]; distance encodings, centrality encodings, and relation-type encodings are injected into the candidate graph. Decision Transformer-based generation (DT-Gen) follows the Goal-Conditioned Decision Transformer for multi-objective robot offline reinforcement learning [31]; task graph generation is reformulated as sequence generation conditioned on desired task return, observation embeddings, and historical graph segments. The POMCP-style planner follows constrained POMDP online Monte Carlo tree search (MCTS) [32]; it performs online tree search over the current belief state and uses the resulting high-value task structure as a strong planning reference.

All baselines use the same candidate task set, observation input, resource-feasibility checker, and evaluation scripts. If an original method lacks resource constraints or a graph-relation prediction head, only necessary input–output adapters are added. These adapters do not include the proposed multi-step teacher distillation, uncertainty gating, or structural repair mechanisms.

### 4.3. Training Convergence and Data Efficiency

Figure 5 reports training curves for task-graph utility, task-edge F1, executable-graph ratio, unseen-policy utility, loss decomposition, and labeled-data efficiency.

Figure 5a–c show that the proposed method rises at a rate comparable to Graphormer and DT-Gen in early training but reaches a higher plateau in the middle and late stages. The final task-graph utility is around 0.85, approximately 3%∼5% higher than the Graphormer training plateau, with similar trends for task-edge F1 and executable-graph ratio. The advantage is traced to the supervision source: while Graphormer captures static candidate-graph relations, it mainly relies on local structural statistics. Under high observation pressure, locally similar geometries correspond to different task meanings. The proposed teacher includes multi-step belief rollout, allowing node and edge labels to reflect future executability rather than the current frame alone.

Figure 5d indicates that the proposed method preserves high utility under unseen adversarial strategies. Figure 5e shows simultaneous decreases in node, relation, value, and resource-penalty losses, suggesting stable joint convergence between structural correctness and executability. Figure 5f further shows that the proposed method approaches a high validation utility even with a moderate number of labeled task graphs, demonstrating the high information density of the search-teacher labels.

### 4.4. Final Performance Distribution and Strong-Baseline Comparison

**Figure 6:**
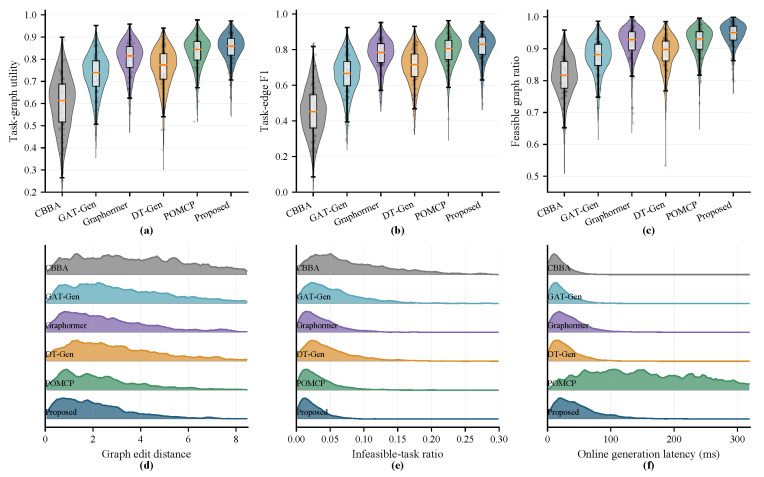
Task graph generation performance on the final test set: (**a**) task-graph utility; (**b**) task-edge F1; (**c**) executable-graph ratio; (**d**) graph edit distance; (**e**) infeasible-task ratio; (**f**) online generation latency.

Figure 6 gives the distributional results for task-graph utility, task-node F1, task-edge F1, executable-graph ratio, graph edit distance, and online generation latency.

Table 8 summarizes the mean performance. The proposed method reaches an average task-graph utility of 0.851, improving over POMCP by 2.24% and over Graphormer by 5.83%. Its task-node F1 and task-edge F1 are 0.892 and 0.815, improving over Graphormer by 5.52% and 5.41%, respectively. The executable-graph ratio reaches 0.945, while the infeasible-task ratio is reduced to 0.024, corresponding to 25.80% and 33.65% reductions relative to POMCP and Graphormer, respectively. In graph edit distance, the proposed method reduces the distance by 13.90% compared with POMCP and by 20.67% compared with Graphormer.

The distributions in Figure 6 show that the proposed method not only improves the mean but also shortens the low-utility tail. POMCP approaches the proposed method in utility, but its average latency is 232.18ms, whereas the proposed method reduces latency by 79.34%. This indicates that POMCP relies on extensive online expansion, while the proposed method compresses planning experience into a neural generator through offline teacher distillation. CBBA and GAT-Gen are faster, but their task-edge F1 and graph edit distance are substantially worse, indicating that fast task selection or local attention alone is insufficient for multi-relation task graphs in adversarial scenarios.

### 4.5. Response Under Complex Situations

**Figure 7:**
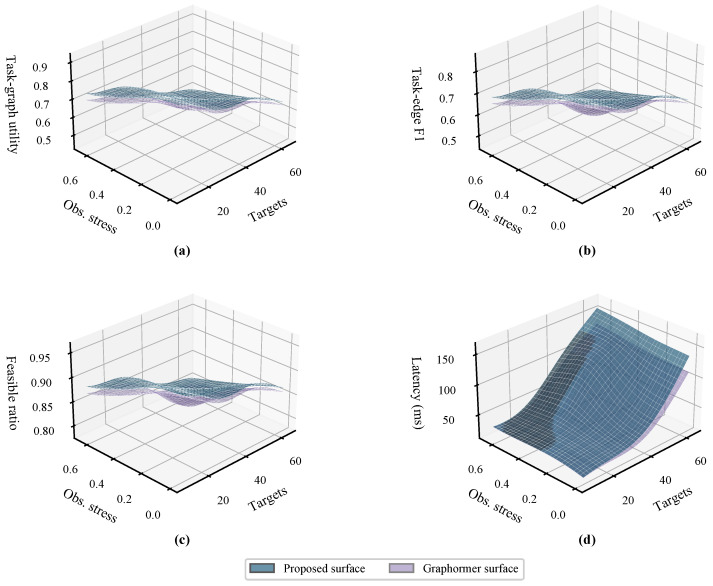
Three-dimensional response surfaces under target scale and observation pressure: (**a**) task-graph utility; (**b**) task-edge F1; (**c**) executable-graph ratio; (**d**) online generation latency. Each panel contains the surfaces of the proposed method and Graphormer.

Figure 7 compares the proposed method and Graphormer under jointly varying target scale and observation pressure.

Figure 7a–c show that both methods degrade as the number of targets and observation pressure increase. This behavior is consistent with partially observable adversarial operation. The proposed method remains above Graphormer over most of the surface, especially in high-scale, high-pressure regions where the executable-graph ratio decreases more slowly. Figure 7d shows that the proposed method is slower than Graphormer but remains far below POMCP, which requires large-scale online tree search.

### 4.6. Paired Statistical Evidence

**Figure 8:**
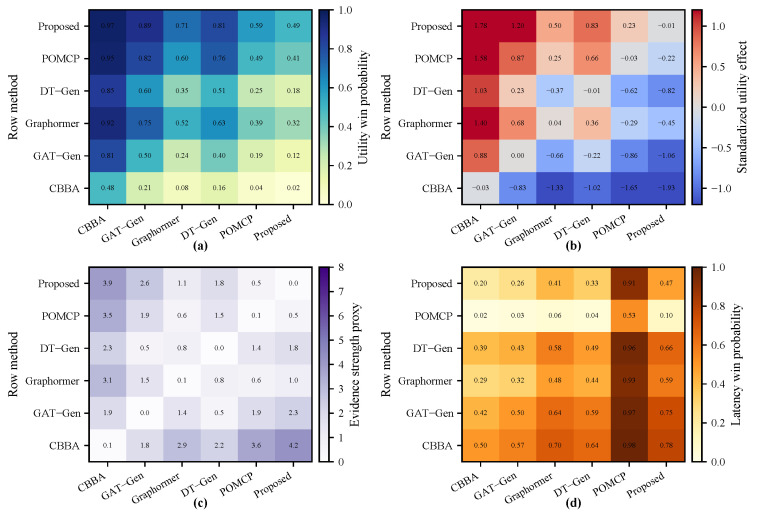
Paired statistical evidence for task graph generation: (**a**) paired win rate in task-graph utility; (**b**) standardized effect size; (**c**) evidence-strength proxy; (**d**) online-latency win rate. Rows and columns denote pairwise method comparisons.

Figure 8 provides paired statistical evidence through utility win rates, standardized effect sizes, evidence-strength proxies, and latency win rates.

The proposed method shows high utility win rates over CBBA, GAT-Gen, DT-Gen, and Graphormer. Its advantage over POMCP is smaller but remains positive, confirming that the improvement does not rely on weak baselines. The effect-size and evidence-strength panels further indicate that the proposed method is most advantageous over conventional task allocation and generic graph generation models, while its advantage over a strong online planner is more moderate. The latency results show a complementary trade-off: CBBA, GAT-Gen, and DT-Gen are faster but structurally weaker, whereas POMCP is structurally strong but computationally expensive. The proposed method achieves near-planner-quality task structures at neural-generator-level latency.

### 4.7. Teacher Budget, Prediction Horizon, and Data Efficiency

**Figure 9:**
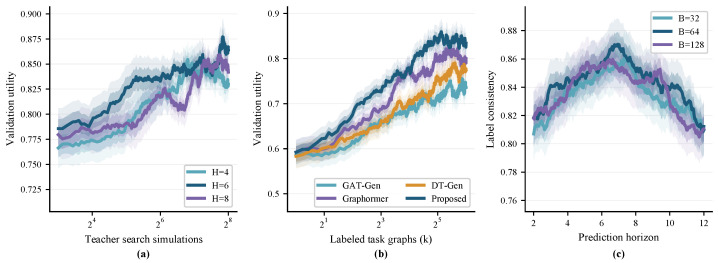
Effects of teacher search budget, labeled data size, and prediction horizon: (**a**) teacher search budget versus validation utility under different horizons; (**b**) data efficiency with different numbers of labeled task graphs; (**c**) prediction horizon versus label consistency under different search budgets.

Figure 9 analyzes the influence of teacher search budget, labeled data size, and prediction horizon.

As the search budget increases from 8 to 256, validation utility first rises rapidly and then saturates. The horizon H=6 performs best overall; H=4 is slightly lower, while H=8 does not yield stable additional gains at high budgets. Increasing labeled task graphs improves all learning methods, but the proposed method reaches the high-utility region with moderate data. These results show that the teacher must provide causal supervision over a suitable horizon. Short horizons miss future values of support, suppression, and confirmation tasks, whereas overly long horizons mix multiple possible futures and make the teacher labels more dispersed.

### 4.8. Uncertainty Modeling and Success-Rate Correlation

**Figure 10:**
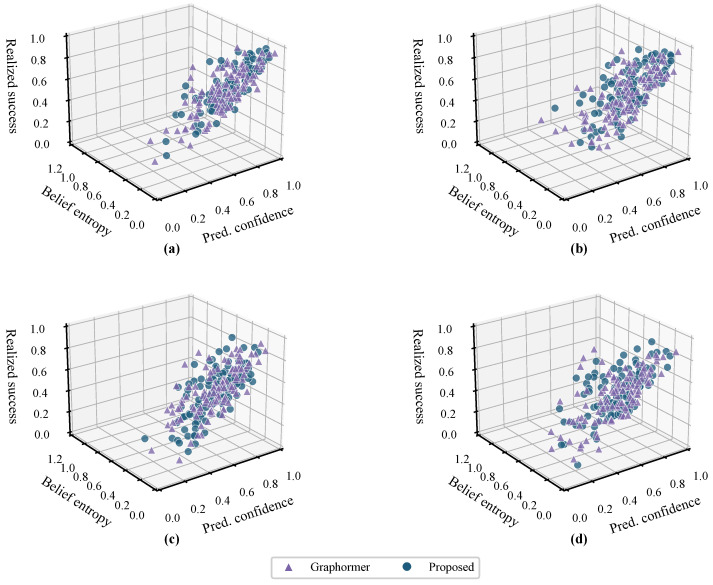
Three-dimensional relation among confidence, belief entropy, and true success rate: (**a**) clean observation; (**b**) moderate noise; (**c**) strong jamming; (**d**) unseen mixed strategy. Each point denotes a generated task graph sample.

Figure 10 visualizes the relation among prediction confidence, belief entropy, and true success rate under clean observation, moderate noise, strong jamming, and unseen mixed strategies.

In this experiment, belief entropy refers to the normalized Shannon entropy of the target-mode belief. For target $e_{j}$, it is computed as

(69) $$H_{j}^{\pi }\left(t\right)=-\frac{1}{\log|M|}\sum_{m\in M}\pi_{j,m}\left(t\right)\log\left(\pi_{j,m}\left(t\right)+\epsilon_{H}\right),$$

where $\pi_{j,m}\left(t\right)$ is the belief probability of mode *m*, |M|=4, and $\epsilon_{H}$ is a small numerical constant. The sample-level belief entropy used in Figure 10 is the mean over active targets,

(70) $$H_{b}\left(t\right)=\frac{1}{M_{t}}\sum_{j=1}^{M_{t}}H_{j}^{\pi }\left(t\right).$$

Thus, $H_{b}\left(t\right)\in \left[0,1\right]$ in the normalized case; larger values indicate more ambiguous target-mode beliefs and therefore greater uncertainty in task selection.

Under clean observations, both the proposed method and Graphormer produce compact point clouds, but the proposed method achieves higher success rates in high-confidence regions. As noise and jamming increase, Graphormer’s point cloud spreads, and some high-confidence samples correspond to low success rates, indicating overconfidence. The proposed method also becomes more dispersed, but high-entropy samples are assigned lower confidence more clearly, preserving a better monotonic relationship between confidence and success rate. This behavior is caused by uncertainty gating and resource-feasibility checking: uncertain nodes tend to be routed to confirmation, tracking, or communication-support tasks rather than high-risk interception tasks.

### 4.9. Candidate Graph Complexity

**Figure 11:**
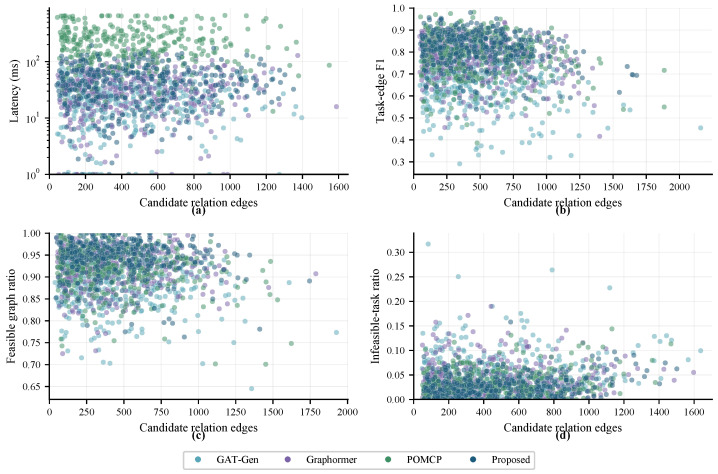
Impact of candidate-graph complexity: (**a**) candidate relation edges versus online latency; (**b**) candidate relation edges versus task-edge F1; (**c**) candidate relation edges versus executable-graph ratio; (**d**) candidate relation edges versus infeasible-task ratio.

Figure 11 analyzes how candidate relation edges affect structural quality and online cost.

As the number of candidate edges increases, all methods experience higher latency and lower task-edge F1 and executable-graph ratio. POMCP is most sensitive because the branching factor of its search tree grows rapidly with relation combinations. Graphormer grows more mildly in latency, but its task-edge F1 drops more sharply in high-complexity regions. The proposed method also becomes slower as complexity grows, yet its samples remain concentrated in regions with a higher task-edge F1 and lower infeasible-task ratio. This suggests that the main difficulty of task graph generation lies not only in the number of nodes but also in the combinatorial explosion of relation edges.

### 4.10. Generalization to Unseen Adversarial Strategies

**Figure 12:**
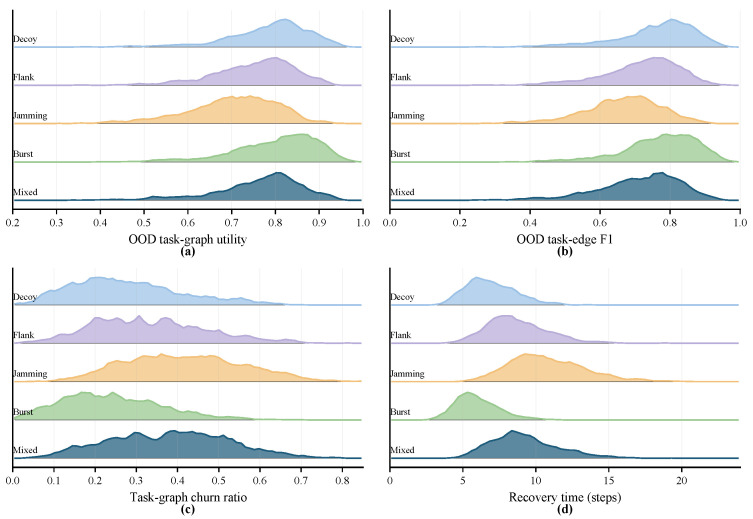
Task graph generation under unseen adversarial strategies: (**a**) task-graph utility; (**b**) task-edge F1; (**c**) task-graph structural change rate; (**d**) recovery steps from structural disturbance.

Figure 12 evaluates task graph generation under red-side strategies unseen during training.

Flank maneuvering and strong jamming reduce both task-graph utility and task-edge F1, while mixed strategies produce the widest distributions, indicating larger structural uncertainty. The proposed method maintains better distributions because task generation is conditioned on belief states that include target motion, communication quality, decoy credibility, and resource margins. When an unseen strategy appears, the generator first detects changes in observation residuals and belief entropy and then reconstructs the graph by adjusting task types and relations. For example, strong jamming encourages communication recovery and target confirmation, while flank maneuvering increases lateral interception and preceding reconnaissance relations.

### 4.11. Tail Risk

**Figure 13:**
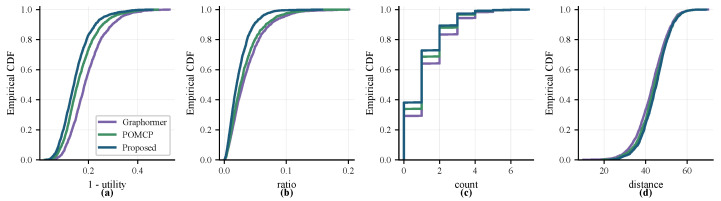
Tail−risk analysis of generated task graphs: (**a**) empirical cumulative distribution of task-generation loss; (**b**) infeasible-task ratio; (**c**) number of structural failures; (**d**) minimum defense margin.

Figure 13 uses empirical cumulative distribution functions to analyze the generation loss, infeasible-task ratio, structural failures, and minimum defense margin.

The proposed method shifts the curves in Figure 13a–c to the left, indicating fewer episodes with high loss, a high infeasible-task ratio, and many structural failures. In Figure 13d, it accumulates faster in larger defense-margin regions, suggesting that it retains more safety buffer in difficult episodes. The reduction of tail risk stems from the suppression of bad structures. Severe failures in adversarial scenarios are often triggered by a few erroneous edges, such as linking decoy confirmation directly to fire interception or generating high-bandwidth task chains under communication disruption. Explicit resource-margin checks, precedence reasoning, conflict edges, and repair of high-uncertainty edges reduce the chance that a local relation error propagates into an infeasible graph.

### 4.12. Ablation Study

**Figure 14:**
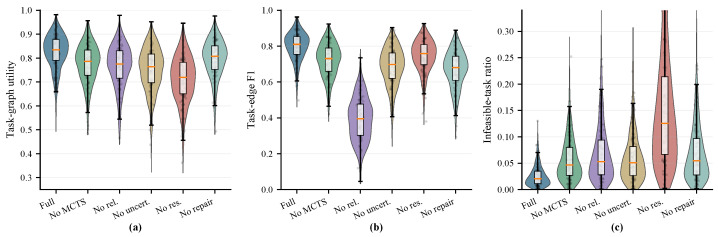
Ablation study of key modules: (**a**) task−graph utility distribution; (**b**) task−edge F1 distribution; (**c**) infeasible−task ratio distribution.

Figure 14 compares the full model with five ablation variants: removing the multi-step teacher, relation bias, uncertainty module, resource constraints, or edge repair.

Removing the multi-step teacher reduces task-graph utility from about 0.842 to about 0.790, showing that one-step supervision cannot sufficiently encode the effect of current task structures on future execution. Removing relation bias causes the largest drop in task-edge F1, from about 0.812 to about 0.410, confirming that node recognition and relation recognition are distinct problems. Removing the uncertainty module decreases both utility and edge F1 and increases the infeasible-task ratio, indicating that belief entropy is important for difficult and ambiguous samples. Removing resource constraints raises the infeasible-task ratio to about 0.135, more than five times that of the full model. Removing edge repair causes a milder utility decrease but still increases infeasibility, showing its role in local conflict removal and tail-risk control.

### 4.13. Task Graph Visualization

**Figure 15:**
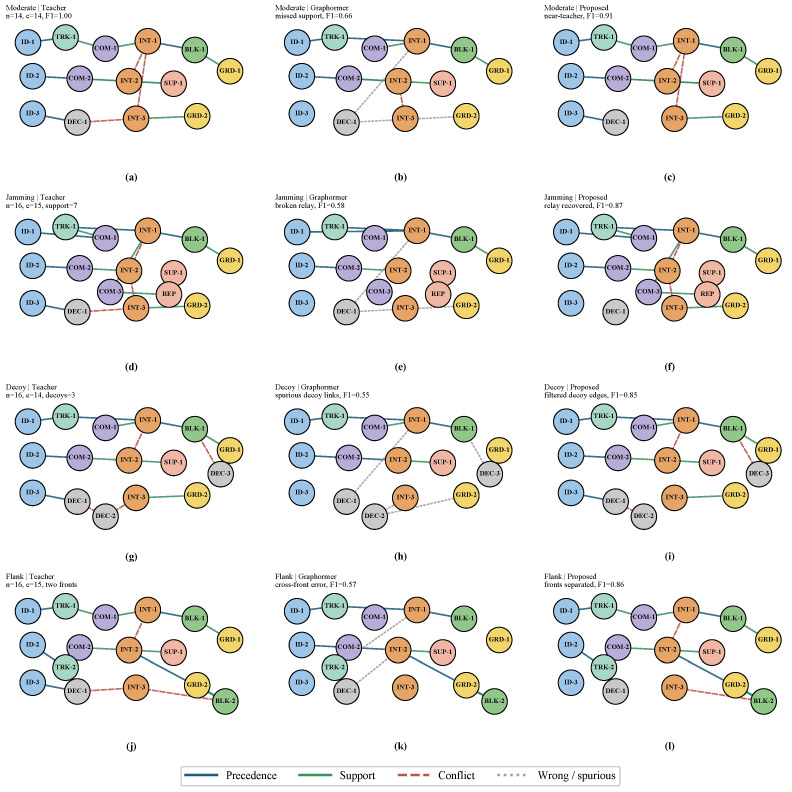
Task graph visualization in typical adversarial scenarios: (**a**–**c**) teacher graph, Graphormer graph, and proposed graph under moderate pressure; (**d**–**f**) strong jamming; (**g**–**i**) decoy deception; (**j**–**l**) flank maneuvering. Node labels denote target identification (ID), tracking (TRK), communication support (COM), interception (INT), blocking (BLK), guarding (GRD), decoy-related tasks (DEC), support tasks (SUP), and relay/repair tasks (REP).

Figure 15 visualizes teacher graphs, Graphormer outputs, and the proposed outputs under moderate pressure, strong jamming, decoy deception, and flank maneuvering.

Under moderate pressure, Graphormer tends to miss support edges, breaking task chains that require communication or confirmation prerequisites; the proposed method reaches a task-edge F1 of about 0.91. Under strong jamming, Graphormer produces relay-chain breaks and obtains a task-edge F1 of about 0.58, while the proposed method restores communication-support relations and increases the score to about 0.87. In decoy-deception scenarios, Graphormer often creates false edges pointing to decoy nodes, whereas the proposed method filters part of these relations through uncertainty and resource-feasibility checks. Under flank maneuvering, Graphormer creates cross-sector erroneous links, while the proposed method tends to preserve separation between different defensive sectors.

## 5. Limitations and Future Validation

The results should be interpreted within the scope of simulation-based evaluation. The simulator includes partial observability, noisy sensing, jamming, decoy behavior, heterogeneous UAV resources, and structural task dependencies, but it does not fully reproduce real flight dynamics, airspace regulations, hardware failures, localization drift, packet-level communication protocols, or safety-certification constraints. The current resource model is also intentionally compact and uses additive consumable-resource accounting, while real missions exhibit nonlinear resource interactions, synergy, contention, and sequence-dependent costs. Consequently, the reported advantages demonstrate observation-conditioned task graph generation under controlled adversarial simulations, rather than direct evidence of operational deployment readiness.

Future validation should therefore proceed in stages. First, measured communication, localization, and energy-consumption traces will calibrate the simulator and replace simplified resource-cost terms. Second, hardware-in-the-loop experiments will test whether the generated precedence, support, and conflict relations remain executable under real onboard computation and communication delays. Third, real or semi-real flight data will evaluate whether belief entropy, uncertainty gating, and task repair remain reliable when the observation model is affected by sensor bias, weather, and platform-specific payload constraints.

## 6. Conclusions

This paper proposed a task graph generation method for heterogeneous UAV swarms in partially observable adversarial environments. The method converts local observations, target beliefs, and platform resources into task graphs with explicit task relations and resource semantics. By combining an offline search teacher, a relation-biased graph attention generator, and an online task manager, it generates task structures that are semantically coherent and executable in the considered simulation setting.

The proposed resource-constrained task graph unifies task types, deadlines, resource requirements, and precedence, support, and conflict relations, enabling generated tasks to reflect both situational demand and execution constraints. The offline search teacher converts future trajectory information, resource feasibility, and structural value into node and edge supervision, reducing the short-horizon limitation of rule-based triggers. The relation-biased graph attention network then learns a fast mapping from candidate task graphs to executable task graphs, supporting online use in the simulator at substantially lower latency than online tree search.

Simulation results show that the proposed method improves task-graph utility, relation prediction, and executability over the compared graph generation baselines. It achieves task-graph quality close to that of POMCP while greatly reducing online generation latency. These findings show that task graph generation serves as a useful front-end task organization mechanism between situation perception and cooperative execution in heterogeneous UAV swarm simulations. Further real-flight and hardware-in-the-loop validation is required before making stronger claims about deployment under hardware, communication, localization, or safety constraints.

## Figures and Tables

**Table 1:** Non-consumable UAV capability dimensions.

Dimension	Capability	Physical Meaning
q=1	Sensing	Target detection, confirmation, and belief correction
q=2	Strike	Interception, blocking, or suppression of red targets
q=3	Communication	Relay, forwarding, and cooperative-link maintenance
q=4	Anti-jamming	Maintaining sensing and communication quality under jamming

**Table 2:** Consumable UAV resource dimensions.

Dimension	Resource	Physical Meaning
h=1	Energy	Flight, maneuvering, hovering, and payload operation
h=2	Strike resource	Ammunition, attack opportunities, or strike windows
h=3	Communication resource	Available links, bandwidth, or relay capacity
h=4	Time resource	Available mission duration or execution window

**Table 3:** Task types and their resource semantics.

ID	Task	Main Capability Requirement	Main Resource Consumption
y=1	Target identification	Sensing, anti-jamming	Low energy and execution time
y=2	Target interception	Strike, sensing	Energy and strike resources
y=3	Area blocking	Strike, persistent presence	Energy and time resources
y=4	Communication support	Communication, anti-jamming	Communication resources and energy
y=5	Boundary guarding	Sensing, strike	Patrol energy and time resources

**Table 4:** Information boundary between the offline search teacher and the online graph generator.

Information Item	Offline Teacher	Online Generator	Usage
Raw observations $O_{t}$	Yes	Yes	Belief update and candidate construction
Belief state $B_{t}$	Yes	Yes	Shared task-generation state
UAV capability and resource states	Yes	Yes	Feasibility and resource-aware scoring
Candidate task graph ${\tilde{G}}_{t}$	Yes	Yes	Teacher labeling and online inference input
Simulated true target state $y_{j}\left(t\right)$	Yes	No	Offline rollout calibration and evaluation only
Future trajectory particles	Yes	No	Search-teacher lookahead labels only
Final execution outcomes	Validation/evaluation only	No	Metric computation and model selection

**Table 5:** Main simulation parameters for task graph generation.

Parameter	Symbol	Range or Setting
Mission area	Ω	1000m×1000m
Number of UAVs	*N*	8∼32
Number of red targets	*M*	6∼48
Candidate task nodes	*K*	8∼64
Candidate relation edges	$|E^{\mathrm{cand}}|$	20∼420
Observation pressure	ρ	0.05∼0.45
Jamming intensity	η	0∼0.5
Prediction horizon	*H*	{4,6,8,10}
Teacher search budget	*B*	8∼256
Labeled task graphs	$|D_{\mathrm{lab}}|$	0.5k∼64k
Evaluation seeds	*S*	10

**Table 6:** Implementation details for the simulation and learning pipeline.

Block	Setting	Value
Dynamics	Decision interval and speeds	Δt=1 step; UAV speed 18∼28m/s; target speed 8∼18m/s
Observation	Sensing and jamming	$p_{0}=0.75$ clipped to [0.05,0.98]; $\sigma_{0}=8\;m$; $R_{\kappa }=180\;m$
Rollouts	Training states	70 episodes × 38 steps = 2660 candidate graph states
Teacher	Lookahead budget	P=22, H=6, and B=128 in the main model
Search	Structural bounds	Beam width 16; $K_{\max}=24$; diversity penalty $\lambda_{\Delta }=0.05$; size penalty $\lambda_{K}=0.08$
Generator	Network	Relation-biased GAT with $L_{g}=4$, d=128, 4 attention heads, and dropout 0.10
Optimizer	Training	AdamW; learning rate $10^{-4}$; weight decay $10^{-4}$; gradient clip 1.0; 10,000 iterations; batch size 48
Loss	Weights	$\lambda_{v}=0.50$; $\lambda_{e}=1.00$; $\lambda_{o}=0.20$; margin Δ=0.15
Evaluation	Test protocol	18 episodes × 75 steps; 10 fixed random seeds for distributional tests
Task manager	Execution thresholds	Node 0.45; edge 0.50; at most 3 tasks per target; at most 24 retained tasks; repair enabled
Baselines	Validation grid	Thresholds and budgets selected on {0.35,0.45,0.55,0.65} with the same resource checker

**Table 7:** Wall-clock cost of data collection, teacher labeling, and generator training.

Pipeline Stage	Workload	Time	Device/Process
Simulator data collection	70 episodes × 38 decision steps	0.42 h	Simulator
Offline teacher labeling	2660 graph states; P=22, H=6, B=128	3.65 h	Search teacher
Generator training	10,000 iterations; batch size 48	1.18 h	RTX 4090 GPU
Validation tuning	10 validation seeds; threshold-grid search	0.36 h	Validation pipeline

**Table 8:** Average performance of the main methods on the final test set.

Method	Utility	Node F1	Edge F1	Executable Ratio	Infeasible-Task Ratio	Latency/ms
CBBA	0.602	0.643	0.452	0.813	0.087	19.46
GAT-Gen	0.731	0.773	0.658	0.874	0.056	23.59
Graphormer	0.804	0.845	0.773	0.920	0.037	36.30
DT-Gen	0.761	0.804	0.705	0.888	0.051	29.40
POMCP	0.832	0.875	0.791	0.922	0.033	232.18
Proposed	**0.851**	**0.892**	**0.815**	**0.945**	**0.024**	47.97

*Note*: Bold values indicate the best result among all compared methods for each accuracy or feasibility metric; latency is reported as a computational-cost metric and is therefore not bolded.

## Data Availability

Dataset available on request from the authors. The raw data supporting the conclusions of this article will be made available by the authors on request.
